# Protein Stability and Unfolding Following Glycine Radical Formation

**DOI:** 10.3390/molecules22040655

**Published:** 2017-04-19

**Authors:** Michael C. Owen, Imre G. Csizmadia, Béla Viskolcz, Birgit Strodel

**Affiliations:** 1 Institute of Complex Systems: Structural Biochemistry (ICS-6) Forschungszentrum Jülich, 52425 Jülich, Germany; b.strodel@fz-juelich.de; 2 Institute of Chemistry, Faculty of Material Science, University of Miskolc, Egyetemváros 1, H-3529 Miskolc, Hungary; icsizmad@rogers.com (I.G.C.); bela.viskolcz@uni-miskolc.hu (B.V.); 3 Department of Chemistry, University of Toronto, Toronto, ON M5S 3H6, Canada; 4 Institute of Theoretical and Computational Chemistry, Heinrich Heine University Düsseldorf, Universitätsstrasse 1, 40225 Düsseldorf, Germany

**Keywords:** protein oxidation, Trp cage, Trp zipper, villin headpiece, oxidative stress, molecular dynamics simulations

## Abstract

Glycine (Gly) residues are particularly susceptible to hydrogen abstraction; which results in the formation of the capto-dative stabilized C_α_-centered Gly radical (GLR) on the protein backbone. We examined the effect of GLR formation on the structure of the Trp cage; tryptophan zipper; and the villin headpiece; three fast-folding and stable miniproteins; using all-atom (OPLS-AA) molecular dynamics simulations. Radicalization changes the conformation of the GLR residue and affects both neighboring residues but did not affect the stability of the Trp zipper. The stability of helices away from the radical center in villin were also affected by radicalization; and GLR in place of Gly15 caused the Trp cage to unfold within 1 µs. These results provide new evidence on the destabilizing effects of protein oxidation by reactive oxygen species.

## 1. Introduction

Due to its role in several pathological disorders and aging, radical-mediated protein oxidation attracts ever-increasing attention [1,2]. The C_α_ radical is prevalent in peptides and proteins because of the capto-dative effect, which occurs when the radical center is between electron-donating and electron-withdrawing groups [3,4]. The hydroxy radical (OH), which can be produced in the cell via the transition metal catalyzed Fenton reaction, is capable of abstracting hydrogen atoms in a site-specific manner [5,6,7,8]. The low bond dissociation energy facilitates the preferential H_α_ abstraction from the C_α_ by the OH radical and other reactive oxygen species (ROS) compared to other sites, though OH addition can also occur [9,10]. The variation in the rate constant (*k*) of OH reactions with amino acid residues range between 10^7^ and 10^10^ M^−1^·s^−1^ [11]. Of the 20 naturally occurring free amino acids, sulfur and aromatic-containing amino acids have the highest reactivity. However, peptides generally react faster than their respective free amino acids, with *k* ranging between 10^8^ and 10^9^ M^−1^·s^−1^ [11]. Furthermore, less reactive ROS may have a higher reactivity to Gly and other C_α_ sites of proteins, which can be attributed to the resulting aforementioned resonance stabilization of the capto-dative effect [10]. Interestingly, the secondary C_α_ radical produced in the case of Gly seems to be more stable than the analogous tertiary radical formed in the other amino acids [11]. Moreover, less reactive radicals preferentially react with Gly and other C_α_ sites of proteins, presumably for the formation of the thermodynamically favored C_α_ radical, as glycyl radicals have been shown to form selectively [12]. In physiology, glycyl radicals have been thought or have been shown to be involved in the catalysis displayed by pyruvate formate lysase, ribonucleotide reductase and benzyl succinate synthase [13,14,15]. Moreover, the production of glycine-extended precursors of peptide hormones by peptidylglycine α-amidating monooxygenase proceeds through the formation of the glycyl radical [16]. More intriguing is the possibility that protein oxidation may play a role in protein misfolding diseases such as Alzheimer′s disease and Creutzfeld–Jakob disease, and that the glycyl radical has been implicated in both of the diseases and may be involved in neurodegeneration [17,18]. Glycyl radicals belong to the a broader group of oxidatively damaged proteins that are usually degraded and eliminated from the cell; however, this process is not efficient and damaged proteins can still accumulate over time [19]. Levels of oxididized, misfolded, and aggregated proteins are also elevated in age-related disorders, such as Alzheimer’s disease (amyloid-β) [20]. Parkinson′s disease (α-synuclein), diabetes (islet amyloid polypeptide), atherosclerosis (apo-B 100) and cataractogenesis (crystallin) [21,22,23]. As outlined in the ′form follows function′ principle of protein structure–function relationships, understanding the structural changes induced by protein oxidation can shed light on how the function of these proteins convert from physiological to pathological states [24].

Several groups have studied the effect of C_α_ radical formation on the structure of peptides and proteins, with a consensus indicating that the planar conformations (φ and Ψ angles close to 0° or 180°) are most favored in residues containing this structure [25,26,27,28]. The glycine radical (GLR) is the most common and forms selectively, as demonstrated in electron paramagnetic resonance and radiolysis studies [4]. Moreover, this residue has been employed in the regioselective photoalkylation of peptides and proteins [29,30]. The glycine radical has also been shown to comprise the active site of pyruvate formate lyase, ribonucleotide reductase and hexylsuccinate synthase [31,32,33].

Despite the wealth of knowledge on the oxidative H_α_ abstraction of the glycine residue and its effect on the structure of peptide analogues, little is known about the effect of hydrogen abstraction on the structure and dynamics of proteins. Molecular dynamics (MD) simulations can provide insight into the dynamic properties of a protein or other systems, by using a classical mechanical energy description of the atoms (or particles) and their interactions as a function of their configuration by means of a force field. These forces are integrated numerically over a time-step, usually on the order of femtoseconds, to yield an atomistic description of the positions and energies of the system over time. This approach is suitable for atomistic analysis of the structural changes that can occur over a relatively short time interval, such as oxidation. A more detailed explanation of MD simulations can be found in reviews [24,33]. Quantum mechanical studies have shown that the planar conformations favored by the φ and Ψ angles of the C_α_-centered radical distorts the potential energy surface of the GLR diamide, which displays a barrier of 60 kJ·mol^−1^ that surrounds the global minimum conformation (φ, Ψ = 180°) [34]. Conversely, hydrogen abstraction from the C_β_ of Ala residues has a negligible effect on the conformation of this residue, as demonstrated in density functional theory (DFT) studies of a penta-alanyl peptide in both the 3_10_-helical and extended conformations [12]. The disparity between the effects of hydrogen abstraction from the C_α_ of the peptide backbone and the C_β_ of the peptide side chain on the conformation of a peptide can be attributed to the proximity of the radical center between the amide bonds which comprise the φ and Ψ angles. The use of the optimized potential for liquid simulations (OPLS) parameters to describe the C_α_-centered radical in Ala (ALR) in MD simulations was used to determine the effect of radical formation on the stability of helical peptides [35,36]. This study demonstrated that radical formation caused the α-helix to unfold in a solvent-dependent manner, and also initiated an α-helix to β-sheet conversion within 250 ns [36]. Further studies of GLR in larger systems, such the well-characterized protein structures used in this work, will continue to elucidate the effects of hydrogen abstraction on protein misfolding and further our understanding on the ability of ROS to denature proteins of physiological significance.

**Figure 1:**
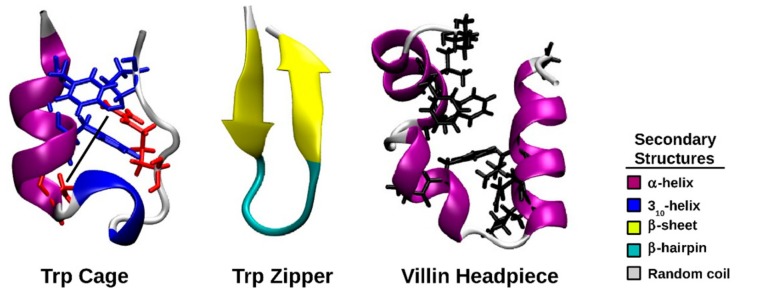
Cartoon representations of the NMR structures of the Trp cage (protein data bank [PDB] code 1L2Y), Trp zipper (PDB code 1LE0), and villin headpiece (PDB code 1YRF). The interacting Tyr3, Trp6 and Pro19 residues are shown in blue, whereas Asp9, Arg16 (shown in red) and the connected salt bridge (dashed line) are also shown. The two leafs of the β-sheet are shown in yellow and the connecting β-hairpin of the Trp zipper is shown in cyan. The residues that form the hydrophobic core of the villin headpiece are shown in black.

The Trp cage is a 20-residue, fast folding mini-protein with an amino acid sequence of NLYIQWLKDGGPSSGRPPPS (PDB code 1L2Y) [37,38]. The PDB structure of the Trp cage indicates that residues 2 to 8 form an α-helix, residues 11 to 14 comprise a 3_10_ helix, whereas residues 15 to 20 adopt a polyproline II structure [38]. The Trp cage is stabilized by a hydrophobic core in which tyrosine and proline surround a Trp residue and a salt bridge is present between Asp9 and Arg16. The Trp zipper is a 12-residue peptide with the primary structure SWTWEGNKWTWK (PDB code 1LE0). A β-hairpin structure is at the center of this protein between the two leafs of a β-pleated sheet [39]. The villin headpiece is a protein domain consisting of three helices and folds on a microsecond timescale. The primary structure of the villin headpiece is LSDEDFKAVFGMTRSAFANLPLWKQQHLKKEKGLF (PDB code 1YRF) [40]. Residues Leu1, Phe6, Val9, Phe10, Ala16, Phe17, Gln25, Lys24, Leu28 and Gly33 form a hydrophobic core with a solvent accessibility of less than 30% [41]. These mini-proteins and their respective characteristics are illustrated in Figure 1. In this study, the effect of peptide backbone radical formation on the structure of these mini-proteins will be studied using molecular dynamics (MD) simulations, using force field parameters derived for the C_α_-centered radical of a Gly residue (GLR) [42]. These parameters were derived using the general method of Lifson and Warshel and yielded energies that were in excellent agreement with those calculated by quantum mechanical methods [42,43]. These proteins were chosen because their structures have been studied by many, and they are stabilized by different structural elements, namely the tryptophan cage, β-sheets and α-helices.

We intend to study the effect of hydrogen abstraction from a single Gly residue from different positions of these proteins. Since it has already been shown both theoretically and experimentally that the Gly residue is easily oxidized and the resulting GLR structure is relatively stable, we endeavor to evaluate how GLR affects the structure of these proteins within the first 100 ns of its formation and compare it to its effect after 1 µs. This work will provide a useful starting point for future studies on free radical initiated protein unfolding, which may include other oxidation sites, mechanisms, and proteins.

## 2. Results

### 2.1. Short-Term Analysis

The dynamics of the first 100 ns of the trajectories of the Trp cage, Trp zipper, villin headpiece and their respective protein radicals are presented as follows.

#### 2.1.1. Cluster Analysis

The results of the cluster analysis of all of the simulated proteins are listed in Table 1. The structures within each cluster were within an RMSD of 1.0 Å of each other. The largest cluster of the closed-shell Trp cage contained 56.6% of the structures in its corresponding trajectory, whereas the largest cluster of the closed-shell Trp zipper and villin contained 97.8% and 63.3% of the structures, respectively. The presence of GLR10 and GLR15 in the Trp cage reduced the cluster size by half, and GLR11 in the villin headpiece led to a reduction by more than two-thirds. The reduction shown by GLR15 in the Trp cage was less pronounced, whereas little change was observed in the size of the largest cluster when GLR6 was introduced in the Trp zipper and GLR33 was introduced in the villin headpiece. The representative Trp cage, Trp zipper and villin headpiece structures obtained from the cluster analysis deviate from their respective NMR structure by 0.86 Å, 0.50 Å, and 2.60 Å, respectively.

**Figure 2:**
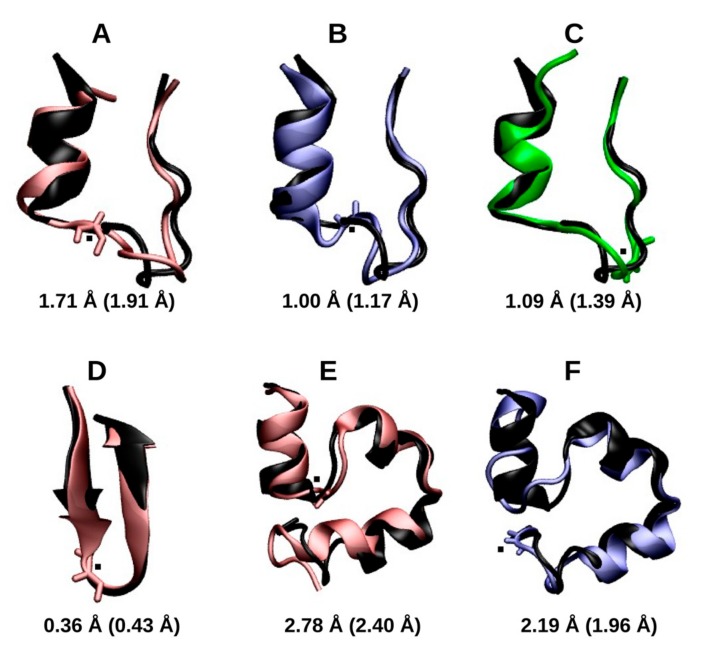
The central structure of the largest cluster in Trp cage C_α_-centered Gly radical in place of Gly10 (GLR10) (in red, **A**), Trp cage(GLR11) (in blue, **B**), Trp cage (GLR15) (in green, **C**), Trp zipper(GLR6) (in red, **D**), villin(GLR11) (in red, **E**), and villin(GLR33) (in blue, **F**). Proteins are aligned with those of their respective closed-shell proteins (in black). The root-mean-squared deviation (RMSD) values of the alignment is shown, whereas the RMSD of each representative structure from the respective starting structure is shown in parentheses. Those of the closed-shell Trp cage, villin, and Trp zipper are 0.86 Å, 0.50 Å, and 2.60 Å, respectively. The residue containing the radical center is shown in the licorice representation, with the radical center represented by a black dot.

The central structure of the largest cluster of each radicalized protein was compared to its respective closed-shell structure. The aligned structures and their respective RMSD values are shown in Figure 2. The RMSD of the radicalized Trp cage Gly residues, GLR10, GLR11 and GLR15, and the RMSD of each radicalized protein with respect to the closed-shell Trp cage is 1.71 Å, 1.00 Å, and 1.09 Å, respectively. In each case, the loop region was distorted; however, the relative positions of the N-terminal helix and the disordered C-terminal region remained unchanged. The RMSD of the radicalized Trp zipper from the structure of the closed-shell protein was only 0.36 Å. Neither the integrity of the β-sheet nor of the turn appears to be disrupted by the radicalization of Gly6. The radicalized villin miniproteins showed the largest deviations from the closed-shell structures. Both the radical at Gly11 and at Gly33 disrupted the helicity of the residues near the radical center, and this affected the relative position of the helices. As result, the villin containing GLR11 had an RMSD of 2.78 Å, whereas villin with GLR33 had an RMSD of 2.19 Å.

**Figure 3:**
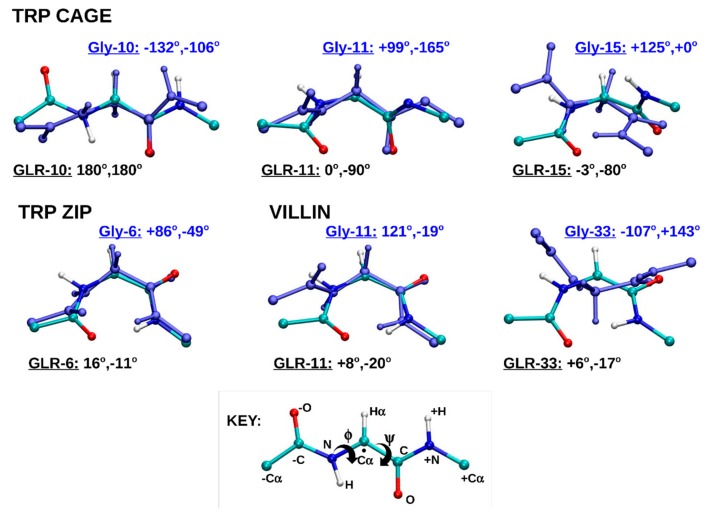
The aligned structures of the each GLR residue of the radicalized protein and its corresponding Gly residue in the respective closed-shell protein. The unpaired electron of the radical center (shown with a dot in the key) is delocalized due to its location between two amide bonds. The atoms with a positive sign (+) belong to the neighboring residue towards the N-terminus, whereas those with a negative sign (−) belong to the neighboring residues towards the C-terminus. The curly arrows indicate the rotated bonds of the φ and Ψ torsional angles.

Figure 3 also compares the central structure of the largest cluster of the simulations; however, in this plot, each GLR residue is aligned to its respective Gly residue. The adjacent amide bonds and the respective Cα atoms of the neighboring residues towards the N-terminus (−C_α_) and the C-terminus (+C_α_) are included in order to provide a closer view of the local (within residue) effect of radicalization. The φ and Ψ angles of each Gly and GLR residue are also shown. The majority of the φ and Ψ angles became planar. GLR10 of the Trp cage became fully extended to φ, Ψ = 180°, 180°. This conformation is also known as *anti*, *anti*, with a reference to the Newman projection of dihedral angles, or β_L_, which is most similar to the conformation of the β-pleated sheet. The GLR residues of the Trp zipper and Villin headpiece each form the C7, or ring conformation, with the φ and Ψ angles close to 0°. This ring is comprised of -O, -C, N, C_α_, C, N+, H+, where the negative sign indicates atoms belonging to the neighboring residue towards the N-terminus and the positive sign indicates atoms belonging to the neighboring residue towards the C-terminus (Figure 3). The conformation of GLR10 of the Trp cage, GLR6 of the Trp zipper and GLR11 and GLR33 of the villin headpiece can all be described as planar, as the atoms in the C7 ring lie (almost) in a plane. This planar conformation is made possible by the loss of an H_α_ atom from Gly when GLR forms, allowing the remaining H_α_ to lie in the plane formed by the C7 atoms.

#### 2.1.2. RMSD and Radius of Gyration

The RMSD-R_gyr_ plot (Figure S1) of the entire closed-shell Trp cage protein indicates that the compactness and structural deviations remained relatively constant during the simulation. The changes to both parameters are less than 2 Å. Fifteen percent of these structures had an RMSD between 2.0 and 2.3 Å and a radius of gyration between 7.0 and 7.3 Å. The distribution of the radicalized Trp cage mini-proteins were similar; however the Gly10 and Gly15 containing structures were divided into two groups, suggesting a transition between two structures of similar stability. The density plot of the radicalized Trp zipper was nearly identical to that of the closed-shell Trp zipper. The radicalization of Gly11 in the villin headpiece reduced the changes in the RMSD of the structure (as shown by the similar sizes of the contour lines), while the RMSD changes increased when Gly33 was radicalized when compared to the closed-shell villin structure. The fact that the densest regions of the three plots are within 0.5 Å of each other indicates that the structures were similar to each other.

#### 2.1.3. Secondary Structure Analysis

**Figure 4:**
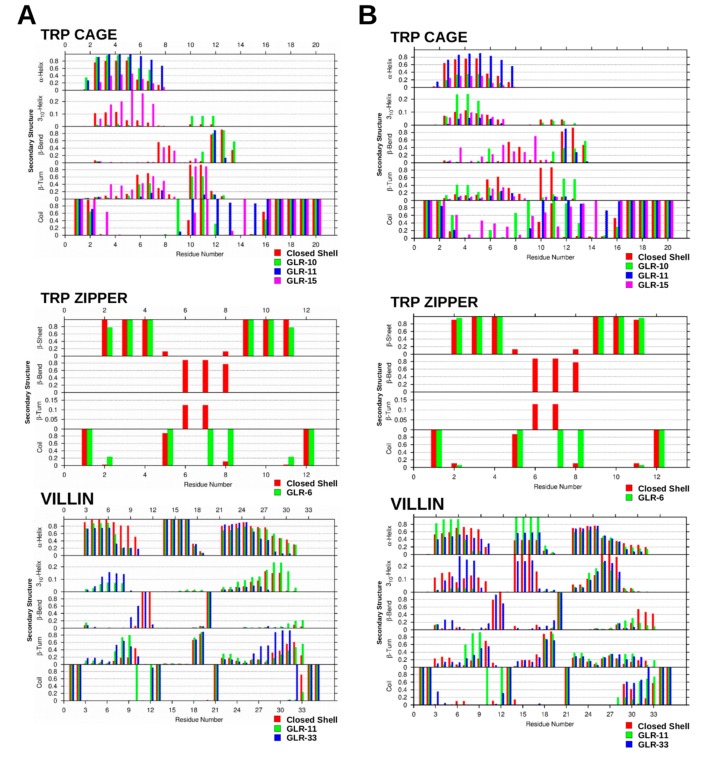
The frequency of secondary structure occurrence at each residue of the closed-shell and radicalized Trp cage, Trp zipper and villin headpiece after 100 ns (**A**) and 1 µs (**B**).

The frequency at which secondary structural elements appear at each residue of the three mini proteins is plotted in Figure 4A. The type of secondary structure recorded depends on its prevalence in each protein, and frequency values greater than 1% are presented. The Trp cage and villin headpiece was comprised of α-helices, 3_10_-helices, β-bends, β-turns, and random coil. The Trp zipper contained a β-sheet but contained no helices. The first and last residue of all three proteins was assigned as a random coil. From the N-terminus to the C-terminus, residue two of the closed-shell Trp cage was also a random coil; however, residues Tyr3 to Gln5 showed greater than 80% α-helicity, which subsequently decreased from 30% to 15% from Trp6 to Lys8. The protein was a mixture of β-turn and β-bend from Lys8 to Gly15, whereas Arg16 to Ser20 were in a random coil.

The helical, turn/bend, and random coil sections observed in the closed-shell Trp cage was also present in the radicalized Trp cage (Figure 4A); however, therein the distribution of the structural elements changes slightly. The presence of GLR10 and GLR11 increased the α-helicity and decreased the β-turn content present in the helical region. Moreover, the radicalization of Gly15 to GLR15 caused the 3_10_-helicity in this region to increase. All three of the GLR residues are within the turn/bend region, and, in each case, the two residues adjacent to GLR were designated as random coil. GLR10 introduced about 8% 3_10_-helicity to this region, but it largely remained a mixture of β-turn and β-bend.

Figure 4A also indicates that the first and last residues of the closed-shell Trp zipper were in the random coil conformation. A β-sheet united Trp2, Thr3, Trp4 with Trp9, Thr10, Trp11, and these regions were on either side of a β-bend. The first residue of the bend (Glu5) was in a random coil, whereas the next three residues were in a β-bend more than 80% of the time, with Gly6 and Asn7 also displaying β-sheet characteristics, and Lys8 showing some random coil properties. When Gly6 is converted to GLR6, the outer residues of the β-sheet (Trp2 and Trp11) converts to a random coil 20% of the time, whereas the β-bridge region is re-assigned to the random coil secondary structure.

The first two and last two residues of the villin headpiece, as shown in Figure 4A, have also been assigned the random coil conformation. The most prominent features of the DSSP assignment of the villin headpiece are the three helical regions, spanning from Ser2 to Phe10, Arg14 to Phe17, and from Leu22 to Glu31. In each region, the α-helicity is highest towards the N-terminus, and decreases towards the C-terminus. The three residues between α-helices one and two are in a β-bend and in a random coil conformation, whereas the four residues between helices two and three are in a β-turn, β-bend and random coil conformation. Both GLR11 and GLR13 reduced the α-helicity of helix one, particularly towards the C-terminus, whilst only GLR33 affected helix three, causing the α-helix content to decrease therein.

#### 2.1.4. Dihedral Angles of Gly and GLR Residues

**Figure 5:**
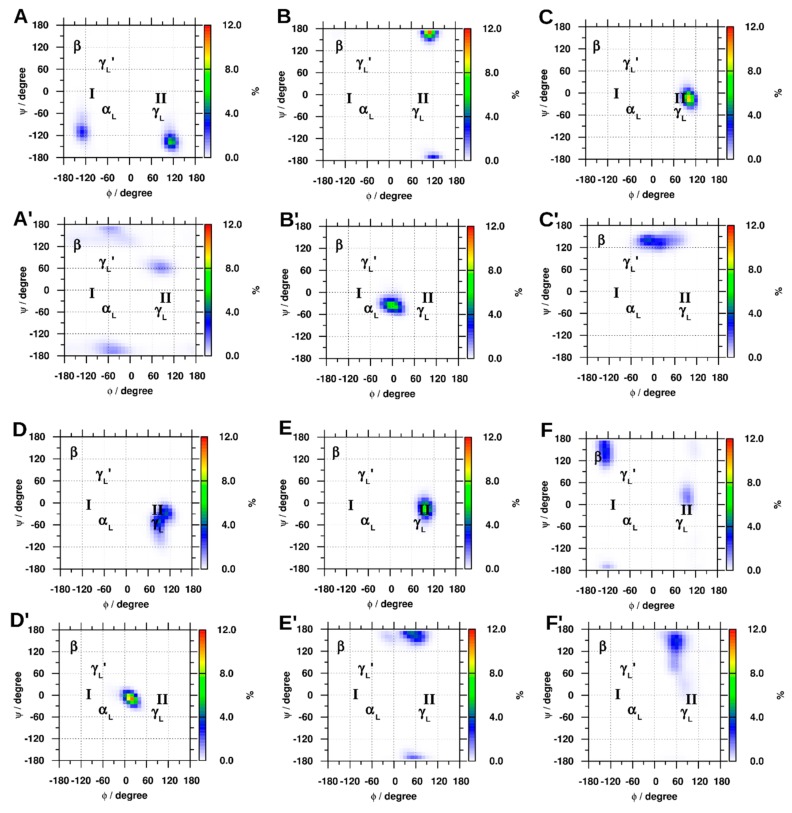
The φ and Ψ density plots for (**A**): Gly10, (**B**): Gly11, (**C**): Gly15 and containing Gly radicals (GLR) at their respective Gly positions (**A′**, **B′**, and **C′**) of Trp cage; (**D**): Gly6 and (**D′**): GLR6 of Trp zipper; along with (**E**): Gly11, (**F**): Gly33 and the respective radicals, (**E′**) and (**F′**) of the villin headpiece. As a reference, the areas of the surface that correspond to the α-helix (α_L_) and β-sheet (β), position two of the classic (γ_L_) and inverted (γ_L_′) γ-turns and position three of type-one (I) and type-two (II) β-turns are are also labeled.

The φ and Ψ density plots in Figure 5 show the regions of the Ramachandran surface that were the most heavily populated by the GLR residues and the respective residue of the closed-shell protein. As a reference, the areas of the surface that correspond to the α-helix (α_L_) and β-sheet (β) are shown. Position two of the classic (γ_L_) and inverted (γ_L_′) γ-turns and position three of type-one (I) and type-two (II) β-turns are often occupied by glycine residues, so these regions are also labeled. In the closed-shell Trp cage, the densest clusters of the φ and Ψ angles of Gly10 were at +120°, −130°, which became 90°, 60° after radicalization. The corresponding angles of Gly11 were +120°, +170° and became 0°, −50° when converting to GLR, whereas the φ and Ψ angles of Gly15 went from a single maximum at +100°, −20°, to a bimodal distribution centered near 0°, +140°. The φ and Ψ angles of Gly6 in the Trp zipper contained a double maximum centered near +100°, −30°, which converted to a single maximum density at +20°, −15°. In the villin headpiece, Gly11 went from +100°, −15° to +60°, +170°, whereas the densest cluster in Gly33 went from −120°, +150° to +60°, +150°.

#### 2.1.5. Hydrogen Bonding

The change in φ and Ψ angles of the affected residues did not have a large effect on the overall structure of the mini-proteins, as judged by the hydrogen bonding observed within the proteins and between the proteins and the solvent water molecules (Figure S2). Seven intrapeptide hydrogen bonds were formed within the closed-shell Trp cage most frequently, which only increased by one when Gly10 and Gly11 were radicalized. A similar difference was observed between the closed-shell and radicalized Trp zipper. However, the largest discrepancy was observed in the villin headpiece, which showed three additional intrapeptide hydrogen bonds when Gly11 was radicalized, and three fewer hydrogen bonds than when Gly33 was radicalized.

### 2.2. Long-Term Effects

**Figure 6:**
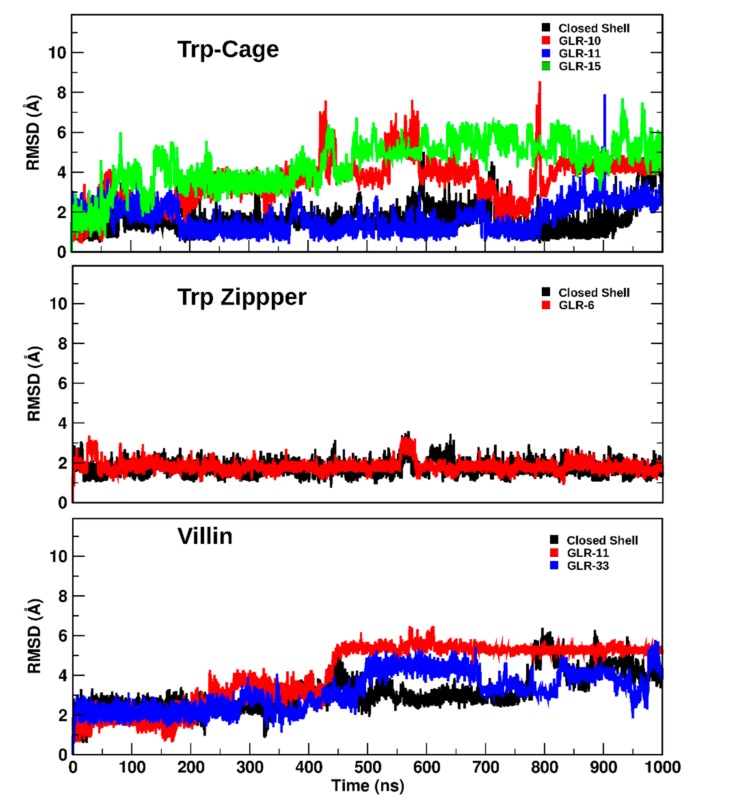
The backbone RMSD as a function of time of the closed-shell and radicalized Trp cage, Trp zipper and villin headpiece.

**Figure 7:**
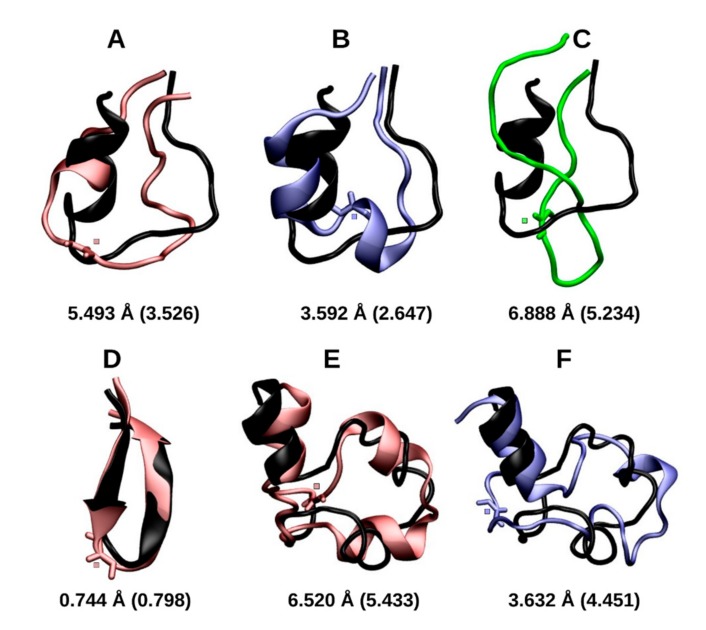
The final structure of Trp cage(GLR10) (in red, **A**), Trp cage(GLR11) (in blue, **B**), Trp cage(GLR15) (in green, **C**), Trp zipper(GLR6) (in red, **D**), villin(GLR11) (in red, **E**), and villin(GLR33) (in blue, **F**). Proteins are aligned with those of their respective closed-shell proteins (in black). The RMSD values of the alignment are shown, whereas the RMSD of each representative structure from the respective starting structure is shown in parentheses. Those of the closed-shell Trp cage, villin, and Trp zipper are 3.885 Å, 1.044 Å, and 4.248 Å, respectively. The residue containing the radical center is shown in the licorice representation, with the radical center.

Exposure of the Trp cage and villin headpiece to the radicalized state for 1 µs further destabilized the protein structure, whereas the 1 µs exposure of the Trp zipper to the radical state had little effect. The backbone RMSD of the closed-shell Trp cage remained within 1 to 2 Å from the starting structure during the first 900 ns (Figure 6) of the trajectory until it subsequently increased to 3.89 Å after 1 µs. This large increase is due to the dissociation of the Asp9-Arg16 salt bridge that stabilized the wild-type Trp Cage dissociated after 920 ns, which caused the broadening of the loop region between the α-helix and polyproline helix. Figure 6 also shows how the structure of the Trp cage containing GLR10 and GLR15 deviated by a larger degree and earlier than the closed-shell structure did, with both structures approaching an RMSD of 4 Å after 250 ns, and 5.49 Å and 6.88 Å after 1 µs, respectively. The deviation of the Trp cage with GLR11 remained similar to that of the closed-shell Trp cage. The final structures for these simulations are shown in Figure 7.

**Figure 8:**
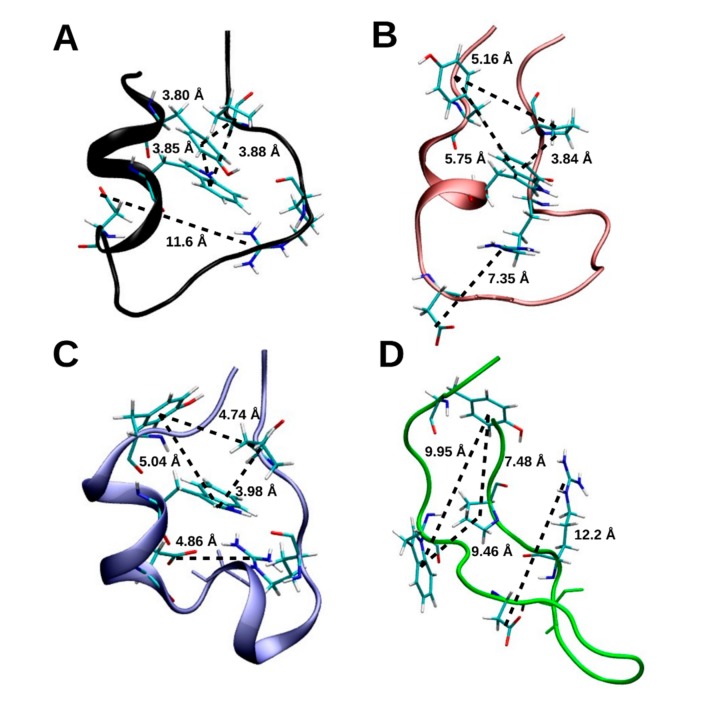
The final structure of the Trp cage-wt (in black, **A**) Trp cage(GLR10) (in red, **B**), Trp cage(GLR11) (in blue, **C**), Trp cage(GLR15) (in green, **D**). The hydrophobic pocket formed by Tyr3, Trp6, and Pro18 remains intact in Trp cage-wt, Trp cage(GLR10), and Trp cage(GLR11), but not in Trp cage(GLR15). The salt bridge between residues Asp9 and Arg16 remains intact in Trp cage-wt and Trp cage(GLR15) but not in Trp cage(GLR10) and in Trp cage(GLR11).

After 1 µs, the Trp, Tyr and Pro residues that from the ‘cage’, after which the protein was named, remained intact in the closed-shell Trp cage (Figure 8). The distance between Trp6 and Pro18, Tyr3 and Pro18, and between Tyr3 and Trp6 in the closed-shell Trp cage was 3.84 Å, 3.72 Å, and 3.92 Å, respectively. However, the distance between Asp9 and Arg16 was 11.6 Å, which indicates that the salt bridge between these residues dissociated.

Secondary structure analysis with the DSSP algorithm indicated no significant changes in the secondary structure of the closed-shell Trp cage when this was compared before and after the 100 ns time point. As shown in Figure 4B, the conformation of residues Tyr3 to Trp6 are more than 80% α-helical, which drops to below 40% after Leu7 and below 20% after Asp9 in the direction of the C-terminus. The residues from Gly10 to Ser13 are largely in the β-bend or β-turn conformation, whereas the latter residues that comprise the polyproline II helix were assigned as a random coil. Despite its deviations, the Trp cage structure remained relatively compact, with the N-terminal α-helix and the C-terminal polyproline II helix remaining largely conserved. The N-terminal α-helical region of the GLR10 Trp cage differed after prolonged exposure to radicalization, which showed an approximately 20% increase in 3_10_-helix propensity at the expense of α-helical structures. This also caused the random coil content of residues 8 and 12 to increase to more than 90%. In the Trp ‘cage’, the distance between between Trp6 and Pro18, Tyr3 and Pro18, and between Tyr3 and Trp6 was 5.16 Å, 3.84 Å, and 5.75 Å, respectively, in Trp cage(GLR10), whereas the same distances were 4.74 Å, 3.98 Å, and 5.04 Å in the Trp cage(GLR11), as shown in Figure 8. The distance between Asp9 and Arg16 in the Trp cage (GLR10) was 7.35 Å, whereas it was 4.86 Å in the Trp cage(GLR11). After 1 µs, the radicalization of Gly11 had no significant effect on the secondary structure of the Trp cage; however, exposure at Gly15 did induce structural changes. The prolonged exposure of Gly15 to radicalization caused an increase in the random coil content of the N-terminal helical region and in the β-bridge/β-turn region. As shown in Figure 7, after 1 µs, the Trp cage(GLR15) is completely unfolded, whereas, in Figure 8, it is shown that all contacts that form the ‘cage’ were lost.

The RMSD of both the closed-shell Trp zipper and Trp zipper(GLR6) remained at approximately 2 Å from that of the starting structure (Figure 6), thus displaying no significant structural changes. After 1 µs, the backbone RMSD between the closed-shell Trp zipper and Trp zipper(GLR6) was only 0.74 Å, with the backbone of the radicalized Trp zipper deviating less from that of the starting structure (0.80 Å) than the closed-shell Trp cage did (1.04 Å), as shown in Figure 7. The exposure of the Trp zipper to radicalization at Gly6 after 1 µs did not result in significant changes to the secondary structures (Figure 4B) that were present during the first 100 ns.

The prolonged exposure of the villin headpiece to radicalization at Gly11 and Gly33 induced more significant changes to those that occurred in either the Trp cage or Trp zipper. After 1 µs, the backbone of the closed-shell villin, villin(GLR11), and villin(GLR33) deviated from the starting structure by 4.23 Å, 5.43 Å, and 4.45 Å, respectively. During the respective simulations, the RMSD of each structure gradually increased until about 450 ns. From then on, villin(GLR11) had the largest RMSD, which remained relatively constant, whereas those of the closed-shell villin and villin(GLR33) oscillated until they reached the aforementioned values after 1 µs. The unfolding of the wild-type villin was initiated by the conversion of the secondary structure from α-helices to β-turns and 3_10_ helices after 420 ns, followed by further displacement of the relative position of the (former) helices, i.e., tertiary structure after 700 ns. (Figure 4B). The exposure of Gly11 to radicalization until 1 µs increased the α-helicity of residues Asp3 to Phe6 and those in the central helical region, whereas Lys7 to Val9 formed a β-turn. On the other hand, the exposure of Gly33 to radicalization until 1 µs elicited a larger decrease in α-helicity than that which occurred in the two other villin structures. The residues adjacent to the radical center in all three mini-proteins was exclusively in the random coil conformation.

## 3. Discussion

The abstraction of hydrogen from amino acid residues is a common mechanism of oxidation by free radicals. Recent studies computed the thermodynamic properties of this reaction for various amino acid residues. Considering the multiple locations from where hydrogen can be abstracted, the ΔE for the reactions can vary. The lowest ΔE for the hydrogen abstraction from the side chain of methionine is −138.7 kJ·mol^−1^, serine is −167.3 kJ·mol^−1^, and asparagine is −183.8 kJ·mol^−1^ [44,45,46]. The ΔE for the reaction of the C_α_ from glycine is comparable to these residues at −144.7 kJ·mol^−1^ [34]. However, hydrogen abstraction from the backbone of Gly has the added consequence of the conformational changes that it causes in peptides, as it has been shown to cause the unfolding of peptide helices and favor the planar (C_7_ and extended) conformations. The effects of H abstraction on the conformation of residues have been done extensively on amino acid diamides [25,26,27,28]. However, to follow-up on a previous study where force field parameters of an alanine radical caused the α-helix to β-sheet conversion of a peptide, this study demonstrated the effect of backbone radicals (GLR) on the structure of proteins for the for the first time [36].

### 3.1. Early Stage Radicalization

As demonstrated by the φ and Ψ angles shown in Figure 5 of the Gly and GLR residues during the first 100 ns of the respective trajectory, the radicalization of Gly11 and Gly15 in the Trp cage and Gly6 in the Trp zipper caused these residues to prefer the planar φ angles of 0°. The Ψ angle of Gly10 residue in the Trp cage, Gly11 of the villin headpiece also converted to planar conformations, whereas the remaining residue, Gly33 of the villin headpiece converted to the ε_D_ conformation, which is the preferred conformation of a d-amino acid residue in the polyproline II helix [47]. Previous DFT studies have shown that the extended β_L_ and the C_7_ ring conformations, both of which are planar, are the most stable conformations of radicals of amino acid diamides [25,26,27,28]. The DSSP analysis indicated that the residues adjacent to GLR residue were unfolded, showing that GLR unfolded the residues of neighboring residues.

Radicalization also had an effect on the secondary structures of residues away from the radical center. As shown in the DSSP analysis during the first 100 ns of the respective trajectory, the radicalization of Gly10 and Gly11 in the β-bend region of the Trp cage caused decreased the helicity of Trp6, Leu7 and Lys8 of the Trp cage helix, whereas the radicalization of GLR15 destabilized the entire helix. The radicalization of both the GLR11 and GLR33 reduced the α-helix content of the N-terminal helix of villin, despite the fact that only GLR11 was adjacent to this helix. Likewise, GLR33 also destabilized the C-terminal helix, wherein this residue is located. The effect of GLR6 on the Trp zipper was less pronounced, though the radicalization of the central residue of the β-hairpin did affect the stability of the outer residues of the β-sheet found in this protein.

These changes in structure were reflected in the cluster analysis completed during the first 100 ns of the trajectory. The size of the clusters were quite large despite the fact that a relatively small clustering cutoff of 1.0 Å was used, which indicated that a high proportion of the structures were within this distance of the central structure. Radicalization reduced the size of these clusters in all but two cases, and yielded clusters of similar as those of the closed-shell protein. However, taking into account the effect of radicalization on the hydrogen bonding, RMSD and the radius of gyration, the tertiary structure of the radicalized mini-proteins remain comparable to their respective closed-shell structure within 100 ns. For example, the structure of the radicalized Trp zipper deviated little from that of the closed-shell structure, as demonstrated by the small RMSD of 0.36 Å. In line with the changes seen in the DSSP plots, radicalization affected the RMSD of the representative structures in the order of Trp zipper < Trp cage < villin headpiece. Moreover, the density plot of the radicalized Trp zipper was nearly identical to that of the closed-shell Trp zipper, with a large overlap between the populated RMSD-Rgyr values. This agrees with the structural similarity of these structures demonstrated in the cluster analysis, DSSP analysis and φ and Ψ angle plots. The radicalization of the individual Gly residues in each mini-protein caused the conformation of the residue to change, however, the overall tertiary structure of the Trp cage and Trp zipper was conserved after each of the Gly residues were sequentially converted to GLR.

### 3.2. Prolonged Radical Exposure

Radicalizaton of the Gly residues of the Trp cage indicated the stabilizing effect of the ‘cage’, formed by the Tyr3, Trp6 and Pro18, and the salt bridge between Asp9 and Arg16. The three structures with the most compact cage, i.e., those with Tyr3, Trp6 and Pro18 distances of approximately 5 Å or less, had the lowest RMSD values, and were able to retain the N-terminal helical, and C-terminal polyproline II structures. Trp cage(GLR15) was the only exception, where the radicalization of Gly15 for 1 µs dissociated the ‘cage’ and unfolded the protein. The salt bridge had a similar stablizing effect, where radicalization of Gly10 made the Trp cage more stable than the closed-shell Trp cage over 1 µs, Moreover, the rise in RMSD of the closed-shell Trp cage after 900 ns corresponded with the dissociation of the Asp9 Arg16 salt bridge. Another example of radicalizaton stabilizing the conformation of a residue can be found in the Trp zipper, where the reduced conformational freedom of the GLR, as shown in quantum chemical studies, stabilized the protein when the φ and Ψ angles of the residue were not far from those of the Gly residue, as shown in Figure 3. Nevertheless, radicalization of the peptide backbone generally destabilized the Trp cage and caused the structure to deviate from its native fold. The effect of Gly radicalization on the destabilization of the Trp cage was in the order of Gly15 > Gly10 > Gly11. It can be seen in Figure 3 that this relation correlates with the deviation of the φ and Ψ angles of each GLR radical from those of its respective Gly residue, with the φ and Ψ angles in Gly15 deviating the most, and those in Gly11 deviating the least. The results were slightly different in the case of the villin headpiece, where the effect of the protein RMSD was greater at Gly11 than at Gly33, but the φ and Ψ angles deviated more at Gly33 than at Gly11 after radicalization. However, the φ and Ψ density plots in Figure 5 show that Gly11 in the closed-shell villin forms a γ-turn (along with Met12), and is also characterized as a turn by DSSP. This turn seems to be important to the structure of the closed-shell villin, as it is disrupted by radical formation and caused large conformational changes in the protein.

### 3.3. General Effects of Gly Residue Radicalization on Stability

The ΔG° of unfolding of Trp cage is +3.2 kJ·mol^−1^, whereas the melting temperature is 317.1 K [48]. The Trp zipper has an unfolding free energy between +2.5 and +7.1 kJ·mol^−1^, and a melting temperature of 323.1 K [42]. The villin headpiece is considered to be ′moderately′ stable in terms of its unfolding free energy (ΔG° = +13.0 kJ·mol^−1^), although it has an experimentally determined melting temperature of 342.1 K and a melting temperature of 325.1 K, as determined in simulations [49,50]. Despite the relatively high melting point with respect to the other two proteins of this study, villin is capable of unfolding and refolding spontaneously [51]. This can account for the large RMSD (4.25 Å) shown by the closed-shell villin in this study. The dissociation of the Asp9-Arg16 salt bridge can account for the relatively large RMSD of the closed-shell Trp cage (3.89 Å); however, the hydrophobic core remained intact and this salt bridge has also been shown to dissociate in simulations with various force fields [52]. The radicalization of the Gly residues affects the RMSD of each protein in the order of villin > Trp cage > Trp zipper, despite the fact that villin has largest ΔG° of unfolding and the highest melting temperature. Only in the case of villin did radicalization cause the RMSD of the respective radical to differ from the closed-shell protein by greater than 2.0 Å after 100 ns; however, the RMSD of radicalized forms of Trp cage and villin increased to almost 7 Å after 1 µs.

After 1 µs, radicalization of GLR15 in the Trp cage and GLR11 in the villin headpiece caused significant unfolding (greater than 5 Å RMSD from the starting experimental structure), whereas radicalization of GLR11 in the Trp cage and GLR33 in villin caused changes that were similar to those observed in the respective closed-shell proteins. Radicalization of GLR10 in Trp cage appeared to stabilize the Trp cage, due to the conservation of the Asp9-Arg16 salt bridge, and also stabilized the Trp zipper when the Gly6 on the hairpin formed a radical. Due to the effects of radicalization on the conformation of the radical center and on neighboring residues, the radicalization of Gly residues has the potential to influence protein function, and initiate complete unfolding within 1 µs as in the case of Trp cage (GLR15).

Experiments have shown that hydrogen bonds contribute 2.1 to 7.5 kJ·mol^−1^ to the energy, whereas theoretical studies say that the energy is closer to zero, due to the canceling out with the hydrogen bond between the protein and water [53]. In this study, radicalization of the protein backbone altered the number of protein–protein hydrogen bonds in each structure, which demonstrates how the removal of one atom can affect the structure of a protein. The removal of a H_α_ from the backbone of Gly6 of the Trp zipper had the lowest effect on the number of hydrogen bonds observed in any of the mini proteins within 100 ns, whereas an increasing number of bonds were affected when an H_α_ was removed from the Trp cage and villin. Using RMSD as a measure of stability, the degree to which the intra-protein hydrogen bonds of each protein were disrupted also shows that these hydrogen bonds affect the stability of these proteins.

## 4. Methods

The molecular dynamics simulations were carried out with the GROMACS 4.5.5 program package (Department of Biophysical Chemistry, University of Groningen. Nijenborgh 4, Groningen, The Netherlands) for the OPLS-AA force field, TIP4P water molecules, and the requisite parameters for the C_α_-centered radical of the glycine residue [42,54,55,56,57,58]. The NMR-determined structures of Trp cage [38]. Trp zipper and the villin headpiece were used as the starting structures for the closed-shell proteins, whereas Na^+^ and Cl^−^ were used as counter ions [39,40]. Each system was energy-minimized by the steepest descent method until the maximum force was less than 100.0 kJ·mol^−1^·nm^−1^. The temperature of the systems was coupled to a modified Berendsen thermostat at 310 K to mimic physiological conditions [59]. Simulations under constant number of particles, volume and temperature (NVT) conditions of the positionally-restrained proteins were performed for 100 ps, followed by a 2 ns simulation under constant number of particles, pressure and temperature (NPT) conditions, at a pressure of 1.0 bar using the Parrinello–Rahman method [60]. These conditions were subsequently employed in each production run, one for each protein, which were 1 µs in duration with a time step of 2 fs. The coordinates of the simulations were saved every 10 ps.

The trajectories were subjected to cluster analysis using the algorithm of Daura et al. with a backbone root-mean-square deviation (RMSD) clustering cutoff of 1.0 Å [61]. The central structure of the largest cluster in each simulation served as the representative structure, which was used to compare the radicalized proteins to its respective closed-shell structure. The structure of each radical center residue (GLR), and its corresponding Gly residue in the respective closed-shell protein were also compared. The occurrence of random coil, bend, turn, α-helix, and 3_10_-helix secondary structure elements during the trajectory was determined using the define secondary structure of proteins (DSSP) algorithm [62]. The frequency at which these elements occurred in each residue was plotted for each of the proteins. The φ and Ψ Ramachandran angles of the GLR residue and those of the corresponding Gly residue of the closed-shell protein were plotted on the Ramachandran surface, which was divided into 10° by 10° subsections. As a reference, the regions of the Ramachandran surface that correspond to the prevalent secondary structures of the Gly residue are also included. The population of the sampled structures were plotted on the RMSD vs the radius of gyration (R_gyr_) surface. The backbone of the respective starting structure was used as a reference for the RMSD calculation. The peptide backbone was used for the R_gyr_ calculation. Finally, the intramolecular and protein-solvent hydrogen bonds of the radicalized and closed-shell mini-proteins were compared.

## 5. Conclusions

In conclusion, these MD simulations have demonstrated that the abstraction of an H_α_ from the backbone of a Gly residue changes the φ and Ψ angles of the residue as well as the conformation of neighboring residues within 100 ns. Radicalization also affected the stability of helices and β-sheets away from the radical center. The hydrogen bonds of the protein backbone are also disrupted, but the changes to the RMSD reveals that the overall fold of the protein generally remains intact during the initial 100 ns. Larger structural changes occurred within 1 µs, including the complete unfolding of the Trp cage when Gly15 was radicalized. In general, the effect of Gly residue radicalization varied and depended on the protein and the position of the radical. There, the β-sheet-containing protein was less susceptible to structural changes, whereas the protein comprised mostly of α-helices was the most susceptible.

These results indicate that a protein that has undergone hydrogen abstraction from Gly residues can exhibit conformational changes, and depending on the position of the Gly residue, this can cause a protein to unfold completely within 1 µs. These structural changes could affect the function of a protein, and perhaps precede prion-like fibril formation, as observed in the aforementioned diseases in which amyloid formation has been linked with oxidative stress. Future studies involving glycyl radical formation on amyloidogenic proteins under aggregation conditions would further support this hypothesis. This work complements the structural changes shown by glycine radicals’ quantum mechanical (QM) calculations and experimental studies and offers new insight into the timescale of the subsequent conformational changes.

## Figures and Tables

**Table 1:** The number of clusters in each simulation of the closed-shell (CS) mini-proteins and the respective radicalized forms resulting from a clustering cutoff of 1.0 Å, the proportion of structures in the most populated cluster and the frame of the middle structure therein.

Structure	Number of Clusters	Proportion of Structures in Most Populated Cluster/%	Frame of Middle Structure of Largest Cluster/ns
CS Trp cage	77	56.6	44.5
Trp cage(GLR10)	81	24.4	89.1
Trp cage(GLR11)	51	43.3	4.0
Trp cage(GLR15)	170	20.7	46.8
CS Trp zipper	6	97.8	63.7
Trp zipper(GLR6)	7	92.6	84.5
CS Villin	71	63.3	44.7
Villin(GLR11)	218	18.3	28.6
Villin(GLR33)	70	68.4	66.3

GLR = C_α_-centered radical of Gly.

## Supplementary Materials

Figures S1 and S2 are available online.
