# Supplementary material for: Protein Stability and Unfolding Following Glycine Radical Formation
Source: Molecules. 2017 Apr 19;22(4):655. doi: 10.3390/molecules22040655 (PMC6154654; doi:10.3390/molecules22040655)
Supplement: Supplementary file 1 [file molecules-22-00655-s001.pdf]

Supplementary Materials: Protein Stability and Unfolding Following Glycine Radical Formation

Michael C. Owen *, Imre G. Csizmadia, Béla Viskolcz and Birgit Strodel

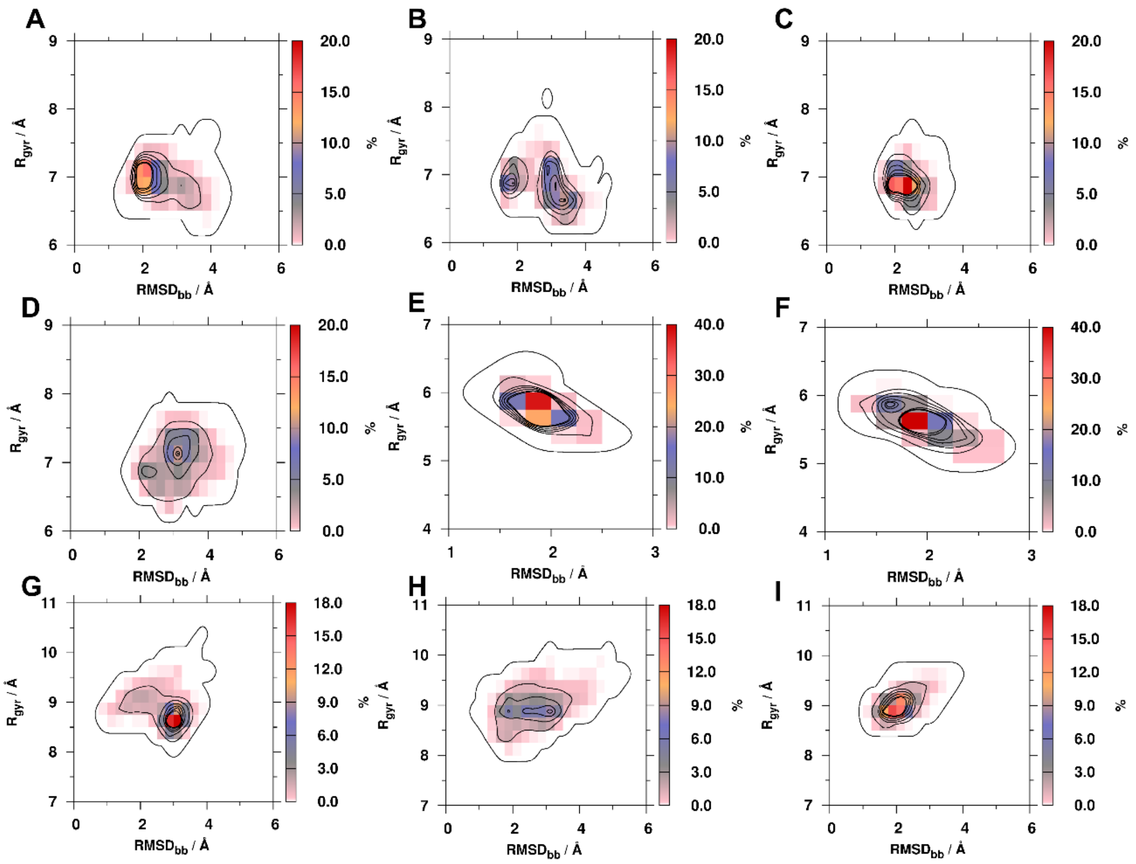

Figure S1. The Rgyr-RMSD density plots of the Trp cage (**A**), Trp cage Cα-centered Gly radical in place of Gly10 (GLR10) (**B**), Trp cage(GLR11) (**C**), Trp cage(GLR15) (**D**), Trp zipper (**E**), Trp zipper(GLR6) (**F**), the villin headpiece (**G**), villin(GLR11) (**H**) and villin(GLR33) (**I**).

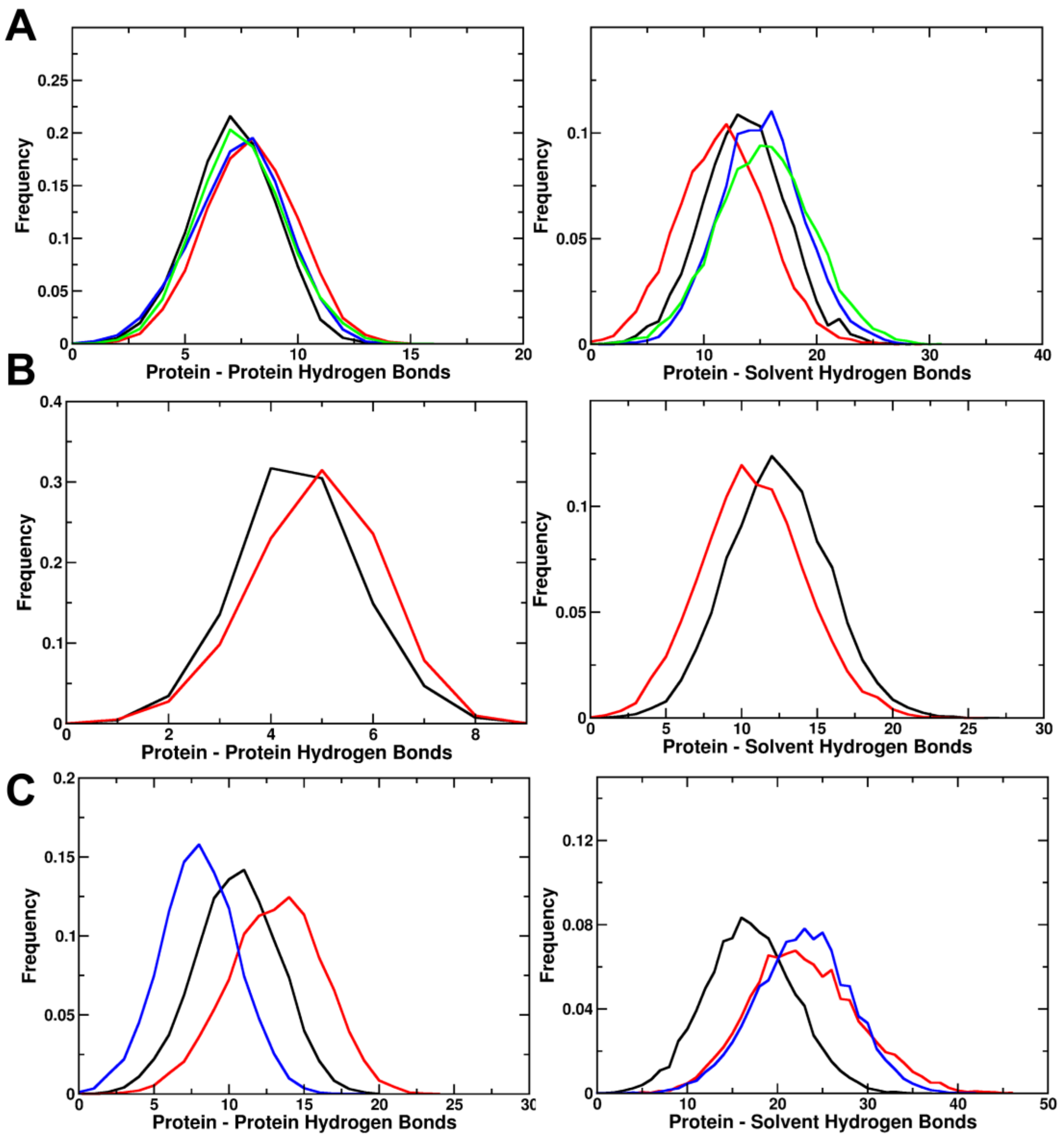

Figure S2. The number of protein-protein hydrogen bonds (left panel) and protein-solvent hydrogen bonds (right panel). Row **A** contains the data for the Trp cage (in black), Trp cage(GLR10) (in red), Trp cage(GLR11) (in red), Trp cage(GLR15) (in blue). Row **B** contains data for the Trp zipper (in black), Trp zipper(GLR6) (in red). Row **C** contains data for the villin headpiece (in black), villin(GLR11) (in red) and villin(GLR33) (in blue).
